# Evaluation of the reducing potential of PSMA-containing endosomes by FRET imaging

**DOI:** 10.20517/cdr.2020.84

**Published:** 2021-03-19

**Authors:** Chelvam Venkatesh, Jiayin Shen, Karson S. Putt, Philip S. Low

**Affiliations:** ^1^Discipline of Chemistry, Indian Institute of Technology, Madhya Pradesh, Indore 453552, India.; ^2^Discipline of Bioscience and Biomedical Engineering, Indian Institute of Technology, Madhya Pradesh, Indore 453552, India.; ^3^Department of Chemistry, Purdue University, West Lafayette, IN 47907, USA.; ^4^Institute for Drug Discovery, Purdue University, West Lafayette, IN 47907, USA.

**Keywords:** DUPA, prostate-specific membrane antigen, endosomes, endocytosis

## Abstract

**Aim**: Ligand-targeted therapeutics are experiencing increasing use for treatment of human diseases due to their ability to concentrate a desired drug at a pathologic site while reducing accumulation in healthy tissues. For many ligand-targeted drug conjugates, a critical aspect of conjugate design lies in engineering release of the therapeutic payload to occur only after its internalization by targeted cells. Because disulfide bond reduction is frequently exploited to ensure intracellular drug release, an understanding of the redox properties of endocytic compartments can be critical to ligand-targeted drug design. While the redox properties of folate receptor trafficking endosomes have been previously reported, little is known about the trafficking of prostate-specific membrane antigen (PSMA), a receptor that is experiencing increasing use for drug targeting in humans.

**Methods**: To obtain this information, we have constructed a PSMA-targeted fluorescence resonance energy transfer pair that reports on disulfide bond reduction by changing fluorescence from red to green.

**Results**: We show here that this reporter exhibits rapid and selective uptake by PSMA-positive cells, and that reduction of its disulfide bond proceeds steadily but incompletely following internalization. The fact that maximal disulfide reduction reaches only ~50%, even after 24 h incubation, suggests that roughly half of the conjugates must traffic through endosomes that display no reducing capacity.

**Conclusion**: As the level of disulfide reduction differs between PSMA trafficked and previously published folate trafficked conjugates, it also follows that not all internalizing receptors are translocated through similar intracellular compartments. Taken together, these data suggest that the efficiency of disulfide bond reduction must be independently analyzed for each receptor trafficking pathway when disulfide bond reduction is exploited for intracellular drug release.

## Introduction

As increasing emphasis is placed on precision medicine, ligand-targeted therapies are becoming increasingly important in treating human diseases^[1,2]^. Advantages of ligand-targeted therapies are manifold, including reduced toxicities to healthy tissues, increased accumulation and retention of drug in pathologic tissues, the capability to administer therapeutic agents that would not be tolerated in non-targeted form, and the ability to select responsive patients using a companion diagnostic comprised of the targeting ligand linked to an imaging agent^[3]^. These targeted therapeutics typically contain a ligand that specifically binds to an upregulated receptor on the pathologic cell surface, a linker region that can be modified to optimize the physical and chemical properties of the conjugate, and a therapeutic payload^[4]^. Unsurprisingly, the design of these conjugates to assure that they remain intact while circulating throughout the body and then reproducibly unload their cargoes after uptake by their target cells is often critical to both safety and efficacy.

While the release of the therapeutic payload of some ligand-targeted drugs is not desired (e.g., radioisotopes), other conjugates require discharge of their payloads to be efficacious^[5]^. For these ligand-drug conjugates, a mechanism that has been frequently exploited for intracellular drug release has involved cleavage of a disulfide bond^[6,7]^. This preference has arisen from the fact that intracellular environments are largely reducing, whereas extracellular environments such as the bloodstream and interstitial spaces are largely oxidizing^[8]^. The above difference in redox potential has been hypothesized to allow for reduction of a disulfide bond within intracellular compartments that would normally remain intact during transit through extracellular environments. Curiously, although the internalization pathways for many cell surface receptors have been characterized (i.e., pinocytosis, clathrin-coated pit mediated endocytosis, potocytosis, micropinocytosis, *etc*.^[9,10]^), little if any information exists on whether all or only a fraction traffic to a reducing environment.

In an earlier report, the intracellular reducing potential of a cancer cell was examined using a folate-targeted conjugate containing a fluorescence resonance energy transfer (FRET) pair in which reduction of a disulfide bond connecting the donor and acceptor dyes led to dequenching of the donor fluorophore and the consequent appearance of its fluorescence^[11]^. Using this strategy, the rates of disulfide bond reduction following conjugate internalization and trafficking through various endosomal compartments were quantifiable. It was found that disulfide bond reduction began immediately upon endocytosis, continued during intracellular trafficking and approached completion by 12 h after administration^[11]^. While this information has proven useful in designing the release of folate-targeted therapeutic agents^[12,13]^, the question has always remained whether the kinetics of disulfide bond reduction in the folate receptor endocytic pathway can be extrapolated to release of ligand-targeted drugs that enter cells and traffic through other endocytic pathways.

As numerous prostate specific membrane antigen (PSMA)-targeted drugs and imaging agents have been studied in both animals^[14-16]^ and humans bearing prostate cancers^[17-19]^, we elected to explore this question using a similar fluorescent FRET pair targeted to prostate cancer cells with a PSMA-targeting ligand. In this brief communication, we show that the PSMA-targeted conjugate is internalized quickly and then traffics in approximately equal amounts through an intracellular compartment in which the disulfide bond is rapidly reduced, i.e., similar to folate-targeted conjugates, or an endosome in which no disulfide reduction occurs.

## Methods

### Materials

H-Cys-2-ClTrt, Fmoc amino acids and amide coupling reagents were purchased from Novabiochem (La Jolla, CA). Solvents were obtained from Sigma Aldrich (St. Louis, MO). RPMI and phosphate buffered saline (PBS) were purchased from Invitrogen (Eugene, OR). Bodipy FL NHS ester reagent, sulforhodamine B, and all other reagents were acquired from ThermoFischer Scientific (Waltham, MA).

### Synthesis of DUPA-peptide linker

The DUPA-peptide linker (DUPA-ACA-Asp-Asp-Lys-Cys-SH) was synthesized using standard Fmoc solid phase peptide synthesis procedures in a standard peptide synthesis apparatus (Chemglass, Vineland, NJ) on H-Cys(Trt)-2-ClTrt resin. H-Cys-2-ClTrt resin (0.12 g, 0.0744 mmol) was initially swelled in 5 mL dichloromethane for 30 min, drained, and swelled in 5 mL dimethylformamide (DMF) 3× for 15 min each. Fmoc-Lys(Boc)-OH (0.087 g, 0.186 mmol), benzotriazol-1-yl-oxytripyrrolidinophosphonium hexafluorophosphate (PyBOP, 0.096 g, 0.190 mmol) and N,N-Diisopropylethylamine (DIPEA, 0.130 mL, 0.744 mmol) in 0.5 mL DMF were added to the peptide vessel containing resin beads and reacted for 6 h. Then, the resin beads were washed 3× with 5 mL of DMF followed by washing 3× with 3 mL of isopropanol. To cleave the NHFmoc protecting group, 3 mL of a 20% piperidine in DMF were added 3× to the peptide vessel. Resin beads then were washed 3× with 3 mL of DMF followed by 3 mL of isopropanol.

Completion of peptide coupling was then confirmed by transferring a few resin beads into a test-tube in which 2 drops each of ninhydrin, phenol, and 0.1% potassium cyanide solution were added. The tube was heated for 2 min at 110 °C and the presence of free amine groups was confirmed by the appearance of a dark blue color on the resin beads (Kaiser test).

A series of amino acids including N-Fmoc-Asp(OtBu)-OH (0.076 g, 0.186 mmol), N-Fmoc-Asp(OtBu)-OH (0.076 g, 0.186 mmol), and N-Fmoc-ACA-OH (0.071 g, 0.186 mmol), and lastly, the Tris (tertiarybutoxy)DUPA [(1,5-dioxopentan-2-yl)ureido-5-oxopentanoic acid] (0.055 g, 0.112 mmol), were all sequentially coupled to the growing peptide chain exactly as described above.

The resin beads were dried for 30 min under a nitrogen atmosphere and the DUPA-peptide conjugate was cleaved from the resin by adding 5 mL of a mixture containing trifluoroacetic acid (TFA), triisopropylsilane, 1,2-ethanedithiol (EDT), and H_2_O in a 9.25:0.25:0.25:0.25 ratio, respectively, followed by incubation for 30 min while bubbling nitrogen. The mixture was evaporated under reduced pressure and the concentrated viscous liquid was precipitated in ice cold diethyl ether. The precipitated, colorless DUPA-peptide linker was dried again under a nitrogen atmosphere and purified via RP-HPLC (20 mmol/L NH_4_OAc buffer, pH = 5.0) using a RP-C18 preparative column (5 µm, 19 mm × 150 mm). Lastly, the acetonitrile was removed under reduced pressure and pure fractions were freeze-dried to yield the DUPA-peptide linker (58 mg, 85%). The molecular mass was determined by LC-MS (+ESI) calculated for [M+H]+ (C36H59N8O18S)+: 923.95 found 923.67.

### Synthesis of the DUPA(Lys)-S,S-sulforhodamine conjugate

The above DUPA-peptide linker conjugate (0.0086 g, 0.00934 mmol) was added to a 5 mL reaction vial containing rhodamine sulfonate (0.005 g, 0.00719 mmol) followed by addition of DMSO (200 µL) and DIPEA (0.013 mL, 0.0719 mmol) under an argon atmosphere. The reaction mixture was stirred overnight and purified by RP-HPLC using 20 mmol/L NH_4_OAc, pH = 7.0, 0-50% CH_3_CN in a 30-min run on a Xbridge (Milford, MA) RP-C18 column (5 µm OBD, 19 mm × 150 mm). The pure HPLC fractions were collected and the acetonitrile was evaporated under reduced pressure using rotary evaporator. The red aqueous solution was lyophilized for 24 h to afford the DUPA(Lys)-S,S-sulforhodamine conjugate (0.0086 g, 78%) as a red solid. The molecular mass was determined by LC-MS using a Waters (Milford, MA) micromass ZQ 4000 mass spectrometer (+ESI) calculated for [M+H]+ (C65H92N11O24S4)+: 1539.74 found 1539.65.

### Preparation of the DUPA-Lys(Bodipy FL)-S,S-sulforhodamine-FRET conjugate

DIPEA (0.007 mL, 0.0389 mmol) was added to a mixture of DUPA-S,S-sulforhodamine conjugate (0.006 g, 0.00389 mmol) and BODIPY FL succinimidyl ester in DMSO (200 µL) in a 5 mL reaction vial and stirred overnight under an argon atmosphere. The reaction mixture was separated by RP-HPLC using 20 mmol/L NH_4_OAc, pH = 7.0, 0-50% CH_3_CN in a 30-min run on a Xbridge (Milford, MA) RP-C18 column (5 µm OBD, 19 mm × 150 mm). The pure HPLC fractions were collected and acetonitrile was evaporated under reduced pressure using rotary evaporator. The red aqueous solution was lyophilized for 24 h to afford the final DUPA-FRET conjugate, DUPA-Lys(Bodipy FL)-S,S-sulforhodamine-FRET conjugate (0.0046 g, 65%) as red solid. The molecular mass was determined by LC-MS using a Waters (Milford, MA) micromass ZQ 4000 mass spectrometer (+ESI) calculated for [M+H]+ (C79H105BF2N13O25S4)+: 1813.81 found 1813.76.

### Analysis of DUPA-FRET fluorophore release following disulfide reduction

The DUPA-FRET conjugate was incubated in the presence or absence of 10-fold molar excess dithiothreitol for 24 h at room temperature. The conjugate and any breakdown products were analyzed via RP-UPLC using a Waters (Milford, MA) Acquity system eluting with 20 mmol/L NH_4_OAc, pH = 7.0, 0-50% CH_3_CN in a 5-min run on a Xbridge (Milford, MA) RP-C18 BEH column (1.7 μm; 2.1 mm × 50 mm).

### Cell culture

LNCaP (PSMA+) and PC-3 cells (PSMA-) were obtained from American Type Culture Collection (Rockville, MD) and grown as a monolayer in 1,640 RPMI medium containing 10% heat-inactivated fetal bovine serum, 1% sodium pyruvate, and 1% penicillin streptomycin in a 5% carbon dioxide: 95% air-humidified atmosphere at 37 °C.

### Binding and internalization of DUPA-FRET

DUPA-FRET (100 nmol/L) was incubated with PC-3 (human PSMA-negative) or LNCaP (human PSMA-positive) cells for 1 h at 37 °C. Cells were washed 3× with PBS and fluorescence was visualized via confocal microscopy. Cells were excited using either a 488 nm or 568 nm laser and fluorescence in both the green and red channels were collected. For kinetic studies, DUPA-FRET (100 nmol/L) was incubated with LNCaP cells for 1 h at 37 °C and then washed 3× with PBS. Cells were then immediately imaged via confocal microscopy or incubated at 37 °C for various times and then imaged. The PBS wash step removes unbound conjugate and allows for internalization resulting in little residual fluorescence on the plasma membrane. Cells were excited using a 488 nm laser and fluorescence in both the green and red channels was collected.

## Results

### Synthesis of the DUPA-FRET conjugate

**Figure 1:**
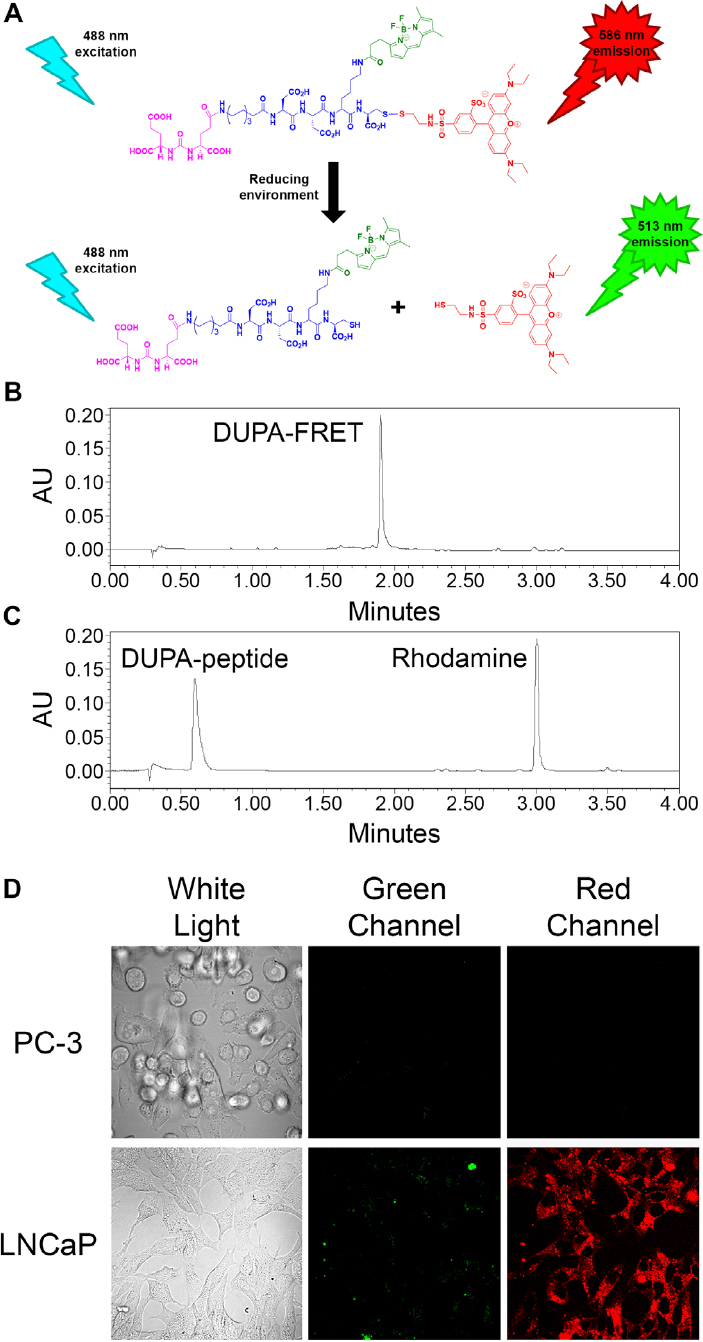
Analysis of disulfide reduction and PSMA binding specificity of the DUPA-FRET conjugate in Scheme 1. (A) Depiction of changes of emitted fluorescence upon DUPA-FRET conjugate reduction. See Scheme 1 for color coding; (B, C) reduction of the DUPA-FRET conjugate was analyzed using RP-C18 HPLC (Abs = 280 nm) in the absence (B) and presence (C) of a 10-fold excess of dithiothreitol; (D) DUPA-FRET conjugate (100 nmol/L) was incubated with PC-3 (human PSMA-negative) and LNCaP (human PSMA-positive) cells for 1 h at 37 °C. Cells were then excited by a green (488 nm) laser and emitted green and red fluorescence were visualized via confocal microscopy

**Scheme 1:**
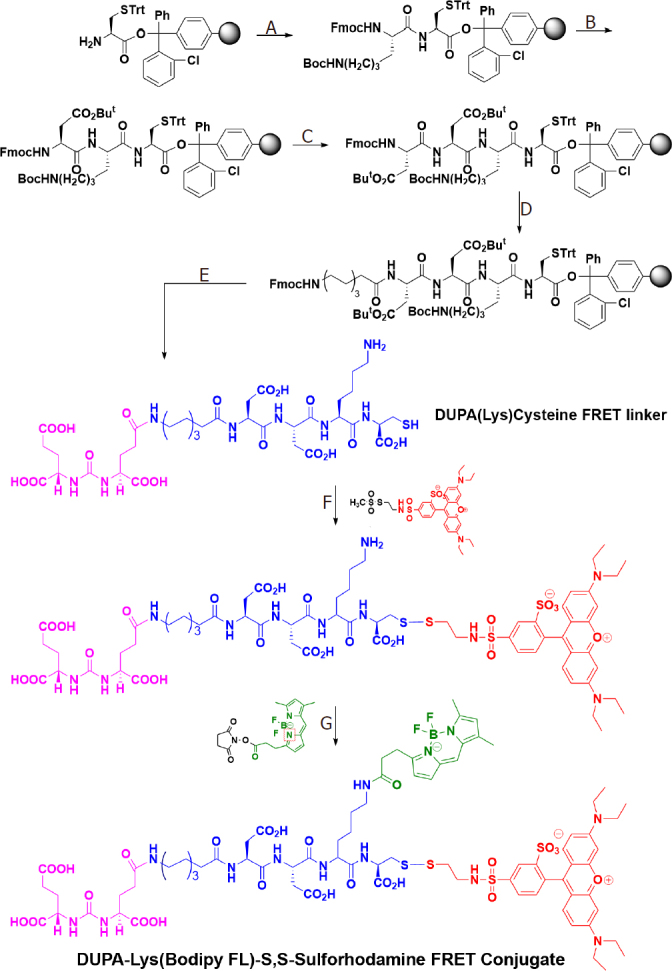
Synthesis of DUPA-Lys(Bodipy FL)-S,S-Sulforhodamine FRET conjugate. Reagents and conditions: (A) Fmoc-Lys(Boc)-OH, PyBOP, DIPEA, DMF, 6 h; (B) (1) 20% piperidine in DMF, rt, 30 min; (2) Fmoc-Asp(O^t^Bu)-OH, PyBOP, DIPEA, DMF, 6 h; (C) (1) 20% piperidine in DMF, rt, 30 min; (2) Fmoc-Asp(O^t^Bu)-OH, PyBOP, DIPEA, DMF, 6 h; (D) (1) 20% piperidine in DMF, rt, 30 min; (2) Fmoc-8-aminocaprylic acid, PyBOP, DIPEA, DMF, 6 h; (E) (1) 20% piperidine in DMF, rt, 30 min; (2) DUPA(O^t^Bu)_3_-OH, PyBOP, DIPEA, DMF, 6 h; (3) TFA/TIS/EDT/H_2_O (9.25:0.25:0.25:0.25) (1 × 5 mL, 30 min; 2 × 5 mL, 5 min); (4) Evaporate TFA; (5) Precipitate in ice cold diethylether, RP-HPLC purification; (F) (1) DIPEA, DMSO, Argon, overnight; (2) RP-HPLC, 20 mM NH_4_OAc, pH = 7.0, 0-50% CH_3_CN in a 30-min run; (G) (1) DIPEA, DMSO, Argon, overnight; (2) RP-HPLC using 20 mmol/L NH_4_OAc, pH = 7.0, 0-50% CH_3_CN in a 30-min run. The final DUPA-FRET conjugate is presented in the bottom structure, where the PSMA targeting ligand (DUPA) is color-coded magenta, the peptidic spacer is color-coded blue, the fluorescence donor (Bodipy) is represented green, and the fluorescence acceptor (sulforhodamine) is colored red

In order to better understand the reducing potential of PSMA-containing endosomes, we constructed a PSMA-targeted disulfide bridged FRET pair, which when excited at 488 nm would emit light at 586 nm prior to disulfide reduction, but 513 nm green light after separation of the FRET pair via disulfide cleavage [Figure 1A]. For this purpose, a PSMA-targeting ligand termed DUPA^[20,21]^ was first attached to an amino acid linker and then sulforhodamine B (ex/em = 565/586 nm) was reacted with the terminal cysteine in the conjugate via a reducible disulfide bond (Scheme 1). Next, Bodipy FL (ex/em = 503/513 nm) was covalently attached via a nonreducible amide bond to yield the desired product.

### Fluorophore release following reduction *in vitro*

Before analysis of the kinetics of disulfide bond reduction in PSMA-containing endosomes, it seemed prudent to demonstrate that the reduction reaction proceeded well under controlled laboratory conditions. For this purpose, the DUPA-FRET conjugate was incubated in the presence or absence of a 10-fold molar excess of dithiothreitol followed by analysis of the reduction products by HPLC. As shown in Figure 1B and C, the conjugate was quantitatively cleaved in the presence of a reducing agent, thus confirming the susceptibility of the disulfide bond to reducing conditions.

### PSMA-mediated internalization of the DUPA-FRET conjugate

Next, to ensure that the DUPA-FRET conjugate would be selectively internalized by PSMA-mediated endocytosis, the conjugate was incubated for 1 hour with either a PSMA-negative (PC-3 cells) or PSMA-positive (LNCaP cells) prostate cancer cell line^[22]^. As shown in Figure 1D, internalization of the conjugate only occurred in the PSMA-positive LNCaP cells, demonstrating that PSMA is essential for DUPA-FRET uptake by prostate cancer cells. Additionally, when the cells were excited with a green (488 nm) laser, mostly red fluorescence was observed, confirming that the conjugate facilitated efficient FRET within the cells.

### Fluorescence changes of the DUPA-FRET conjugate following endocytosis

**Figure 2:**
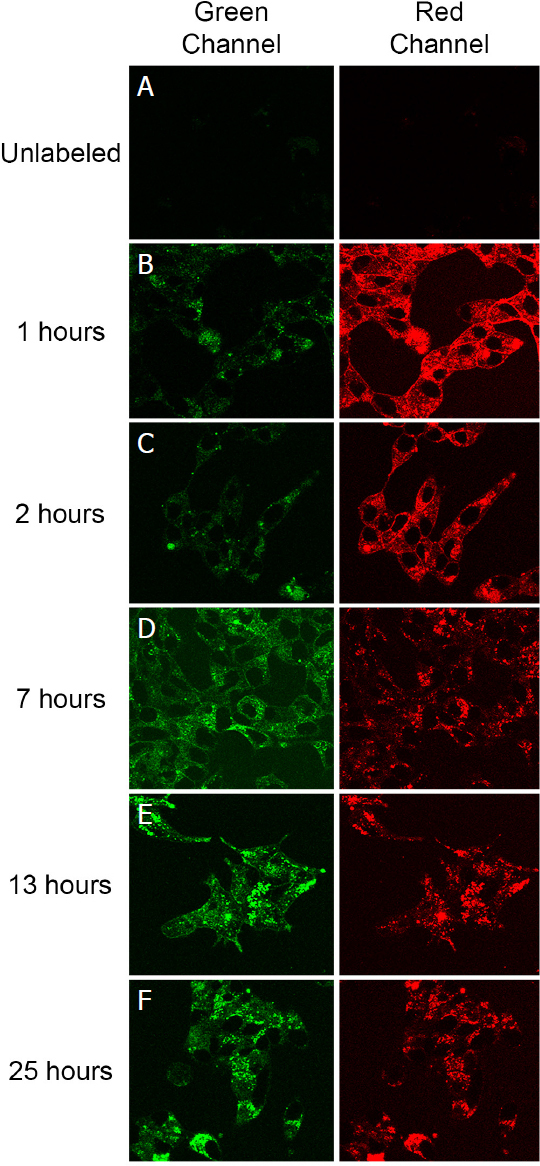
Analysis of the kinetics of disulfide reduction in the DUPA-FRET conjugate following internationalization by LNCaP cells. (A) Cells were either left unlabeled or (B-F) labeled with DUPA-FRET (100 nmol/L) for the indicated times at 37 °C before evaluation by confocal microscopy using 488 nm excitation. A decrease in red fluorescence accompanied by an increase in green fluorescence demonstrates release of the rhodamine from the DUPA-FRET conjugate; (B) image of cells at the 1-h time point showing a punctate distribution of fluorescence throughout the cell interior; i.e., suggesting accumulation of the conjugate within intracellular compartments

**Figure 3:**
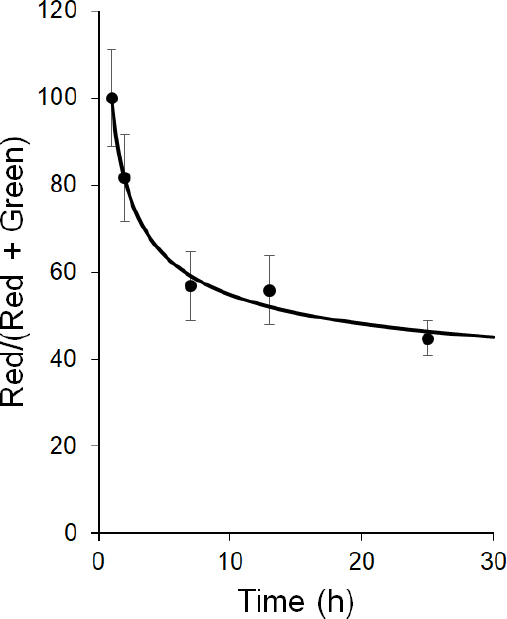
Quantitation of DUPA-FRET fluorescence change upon internationalization in LNCaP cells. Red and green channel fluorescence intensity was quantitated and the percentage of the red compared to total fluorescence was determined and plotted. Error bars represent standard deviation. FRET: fluorescence resonance energy transfer

With the fluorescent properties of the PSMA-targeted DUPA-FRET conjugate confirmed, the reducing potential of PSMA-trafficking endosomes was evaluated by incubating LNCaP cells in the presence of the conjugate for various times and measuring the fluorescent signals from both sulforhodamine B (i.e., red FRET signal of an intact conjugate) and Bodipy (i.e., green signal of a reduced conjugate). As shown in Figure 2, the red FRET signal predominated during the first two hours when the cells were excited at 488 nm, with the vast majority of fluorescence localized to intracellular endosomes even by the 1 h time point (panel 2B). However, as time proceeded, the red FRET signal declined while the green signal increased, indicating that disulfide bond reduction was occurring and the donor/acceptor FRET pair were separating. That the decrease in red fluorescence during 488 nm excitation did not derive from destruction of the rhodamine fluorophore was established by demonstrating that direct excitation of the rhodamine dye with 568 nm light revealed no diminution of fluorescence (data not shown). Interestingly, however, changes in the FRET signal indicated that cleavage of the disulfide bond proceeded to only ~50% completion, even after 24 h incubation [Figure 3], suggesting that approximately half of the DUPA-FRET conjugate must traffic to an endosomal compartment in which the reducing environment is insufficient to cleave a disulfide bond.

## Discussion

In conclusion, information on the internalization and reduction of disulfide bonds can be critical to the design of ligand-targeted drugs, especially in cases where targeting ligand and drug are connected by a disulfide bond^[23,24]^. However, while PSMA is a commonly used receptor for such ligand-targeted therapeutic agents^[14-19,25]^, and recycling of the PSMA receptor has been studied^[26]^, the kinetics of disulfide bond cleavage in PSMA-containing intracellular compartments has not been thoroughly explored. Therefore, in order to measure these kinetics and gain a greater understanding of the reducing environment within PSMA-trafficking endosomes, a PSMA-targeted FRET-based probe containing the DUPA-targeting ligand linked to a disulfide-bridged FRET donor/acceptor pair was synthesized.

This PSMA-targeted drug conjugate was intentionally designed to contain membrane impermeable fluorescent reporter molecules. Thus, the green fluorescent dye is attached to the DUPA ligand via a non-cleavable linker in order to allow the investigator to track where the DUPA ligand traffics following its endocytosis by the cancer cell. Similarly, the red fluorescent dye was also designed to be membrane impermeable to allow the researcher to identify and monitor the endosomes in which the released drug would normally traffic. Thus, we intentionally made our fluorescent reporter molecules membrane impermeable in order to enable us to track their itineraries within the cancer cell. However, if we were to prepare a prostate cancer targeted therapeutic drug, it would be designed to be membrane permeable so that when it was released within an intracellular endosome, it would be able to diffuse out of the endosome and engage its therapeutic target elsewhere in the cell.

When PSMA-positive cells were incubated with this DUPA-FRET conjugate, rapid endocytosis was found to occur, followed by trafficking through intracellular compartments. Although the initial rate of disulfide bond cleavage was fairly rapid (t_1/2_~3 h, Figure 3), the rate plateaued after ~6 h, progressing little further even out to 24 h. In fact, at the 24-h time point, only ~50% of the FRET conjugate had been reduced. When this disulfide cleavage rate is compared to the cleavage rate in folate receptor-containing endosomes the differences are striking. Thus, by 12-h post-incubation with a related folate-targeted FRET conjugate, folate receptor positive KB cells had reduced ~75% of the conjugates and by 24 h post-administration essentially all of the disulfide bonds had been cleaved^[11]^.

In summary, these data suggest that PSMA must traffic through two distinct endosomal processing pathways. Although the associated intracellular processing compartments were not characterized, one must conclude that at least one pathway includes a reducing endosome, while at least one other pathway does not. Because the folate trafficking pathway examined earlier^[11]^ results in quantitative disulfide reduction, a second conclusion must be that not all internalizing receptors traffic through identical intracellular compartments. For ligand-targeted drug delivery, the obvious third conclusion is that the intracellular trafficking pathways that process different ligand-targeted receptors must be characterized before a redox catalyzed cleavage mechanism should be exploited for intracellular drug release.

## Declarations

### Availability of data and materials

All data source could be available to readers on request.

### Copyright

© The Author(s) 2021.
